# High-intensity focused ultrasound rectoanal lifting (HIFU-RAL) for prolapsed hemorrhoids: a case series

**DOI:** 10.1093/jscr/rjaf650

**Published:** 2025-09-20

**Authors:** Shunsuke Suzuki

**Affiliations:** Suzuki Proctology-Moriguchi Internal Medicine Clinic, 16-14 Nasukawa-cho, Morioka, Iwate 020-0016, Japan

**Keywords:** high-intensity focused ultrasound, prolapsed hemorrhoids, HIFU rectoanal lifting, longitudinal muscles, micro-focused ultrasound, minimally invasive treatment

## Abstract

Prolapsed hemorrhoids can affect quality of life, owing to associated pain and other symptoms. Although hemorrhoidectomies are curative, postoperative complications such as pain, bleeding, and delayed recovery are unavoidable. Some patients cannot undergo surgery due to comorbidities or social factors. These challenges highlight the need for minimally invasive, immediate treatments. This study explored the effectiveness of high-intensity focused ultrasound rectoanal lifting (HIFU-RAL) in 10 patients with grade IV hemorrhoids. The procedure was completed within 13.5 ± 3.2 min. Immediately afterward, the prolapsed lesions visibly shrank or disappeared. No prolapse was observed between 9 days and 3 months following HIFU-RAL, and no adverse events were reported. Heating induced by HIFU may have triggered contraction of the longitudinal muscles, repositioning the hemorrhoids to their anatomical location and visually reducing the prolapsed lesion. HIFU-RAL is a promising, minimally invasive treatment for prolapsed hemorrhoids that can be performed immediately in a consultation room.

## Introduction

Prolapsed hemorrhoids are a common anorectal disease that affects physical and mental health, reducing quality of life and often requiring surgery [1, 2]. Hemorrhoidectomies are the most effective treatment; however, postoperative symptoms such as pain and bleeding may persist, affecting recovery and satisfaction [3, 4]. The demand for minimally invasive and immediately effective treatments is, therefore, increasing among patients with physical or social limitations, such as lactating women, anticoagulant users, those with severe comorbidities, and the elderly. High-intensity focused ultrasound (HIFU) is a minimally invasive technique in which ultrasound waves from a focused transducer enter the body, target a site less than 1 mm^3^, induce coagulative necrosis, and prevent surrounding tissue damage [5–8]. HIFU is used to treat prostate cancer, uterine fibroids, pancreatic cancer, breast cancer, skin tightening, genitourinary syndrome of menopause, and urinary incontinence [9–16].

A transanal HIFU method was developed, and during research on fecal and urinary incontinence (FI/UI), hemorrhoid reduction, disappearance, and anal verge elevation were frequently observed following treatment [17]. This report describes the efficacy and safety of HIFU rectoanal lifting (HIFU-RAL) for prolapsed hemorrhoids in 10 cases.

## Case series

### Surgical procedures

**Figure 1:**
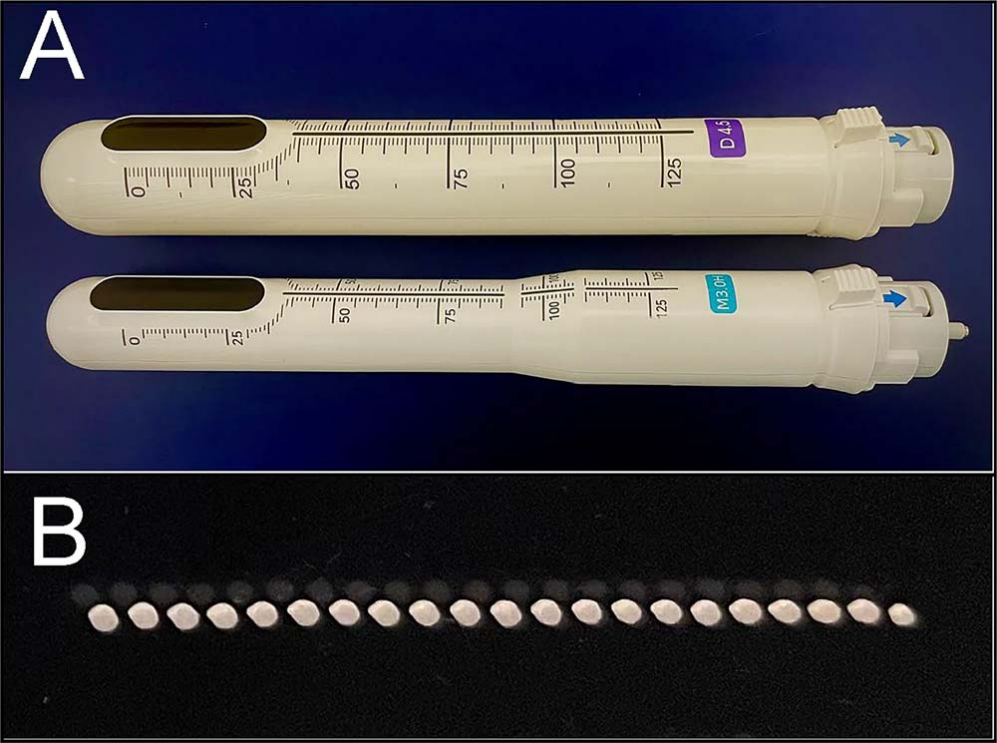
HIRONIC cartridges and irradiation test. (A) Upper transvaginal cartridge (for the vagina), 28 mm in diameter. Lower, newly developed transanal cartridge (for the anus), 23 mm in diameter. (B) Irradiation test. Irradiation was conducted from the back of an acrylic plate under the following conditions: Irradiation range, 25 mm; interval, 1.2 mm; and maximum output, 2.0 J. As a result of heating, all 21 irradiation sites showed degeneration, leading to a noticeable white discoloration.

HIFU was performed using the Ultra Vera™ (HIRONIC Co., Ltd., Gyeonggi-do, Republic of Korea), a device used for vaginal reduction and UI. In Case 1 (our first case), a 28-mm transvaginal cartridge was used. Thereafter, a 23-mm transanal cartridge, developed with HIRONIC, was applied (Cases 2–10; Fig. 1A).

**Figure 2:**
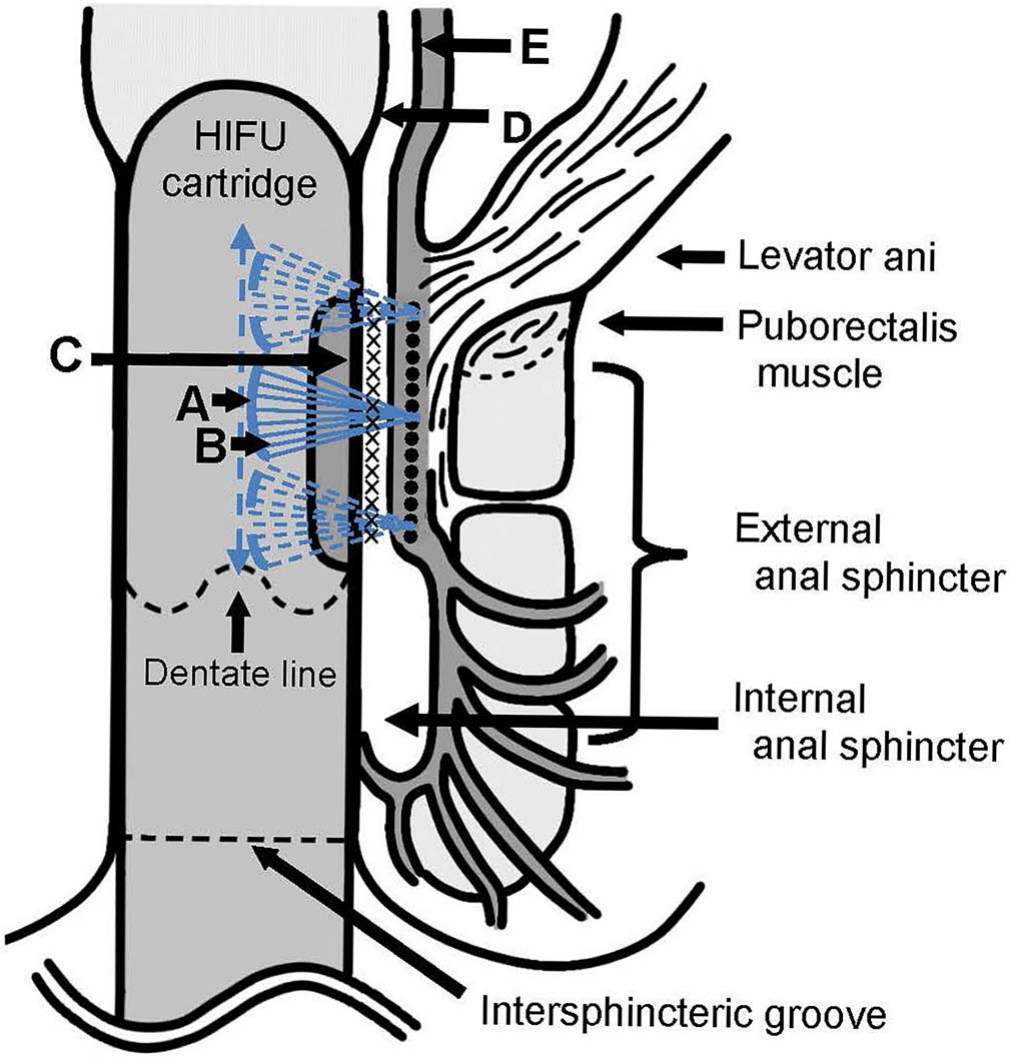
Schematic illustration of the delivery of HIFU. A focused transducer (A) with a curved surface emits ultrasound waves (B) through the vibrations of the piezoelectric materials. These waves are emitted from the emitting surface (C) of the HIFU cartridge and are focused on the irradiation site (**×**) (●). At a focal depth of 3.0 mm, the target was assumed to be the circular muscle and internal anal sphincter, whereas at a focal depth of 4.5 mm, it was thought to be the longitudinal muscle layer.

Two cartridge types were used: D4.5 (4 MHz, max power 2.0 J) and M3.0 (7 MHz, max power 1.2 J), with irradiation depths of 4.5 and 3.0 mm, respectively. The patient was placed in the left lower lateral recumbent position, and the anus was carefully dilated with a silicone dilator to match the cartridge diameter, without anesthesia. The cartridge was inserted until the whole HIFU-emitting surface passed the dentate line, and the entire circumference was irradiated (Fig. 2). The irradiation range was 25 mm, with a 1.2-mm interval (Fig. 1B). Before HIFU treatment, a digital rectal examination and anorectal manometry were performed. Patients with anal stenosis or high maximum resting pressure (MRP) underwent closed lateral internal sphincterotomy (LIS) under local anesthesia immediately before the HIFU-RAL procedure. Anorectal manometry was conducted using a GMMS-100 device (Star Medical Co., Ltd., Tokyo, Japan).

### Cases

**Figure 3:**
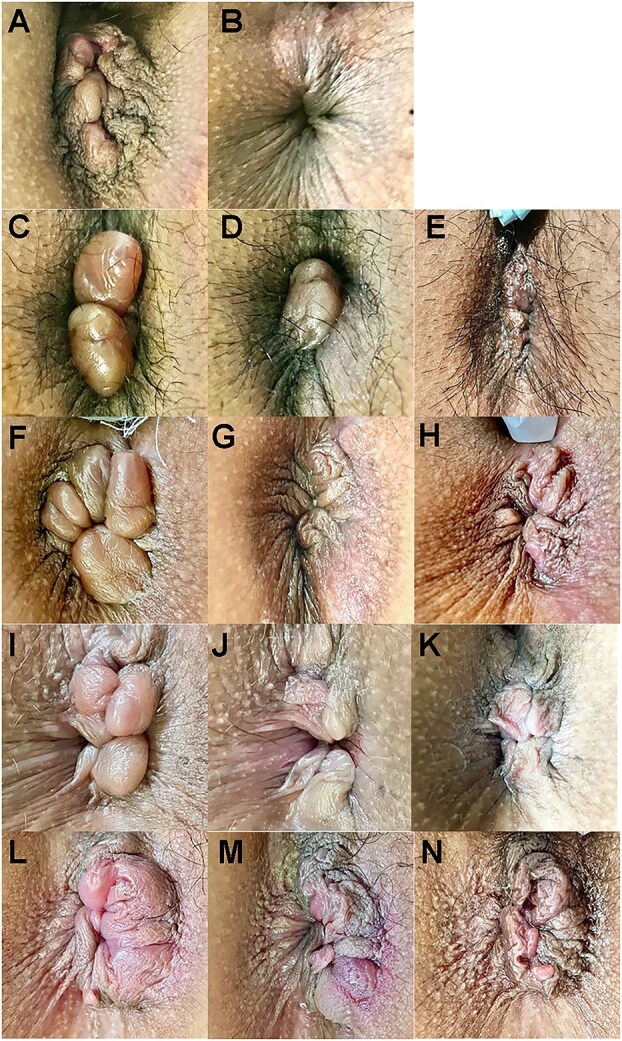
Longitudinal observation before and after HIFU rectoanal lifting (Cases 1–5). Top: Ventral. (A and B) Case 1 before and after HIFU (12 min). (C–E) Case 2 before and after HIFU (11 min) and 9 days later. (F–H) Case 3 before and after HIFU (9 min) and 19 days later. (I–K) Case 4 before and after HIFU (15 min) and 2 months later. (L–N) Case 5 before and after HIFU (16 min) and 15 days later. The numbers in brackets indicate the time elapsed since the photograph was taken before HIFU.

Case 1 involved a 31-year-old exclusively breastfeeding woman (a physician) with a history of hemorrhoid surgery (6 years ago) and a recent spontaneous delivery (Table 1A). Four months postpartum, she developed prolapsed hemorrhoids upon returning to work, presenting with difficulty in self-replacement 1 month later. Since the patient declined medication, HIFU-RAL was performed the following day. The prolapsed hemorrhoids with edema (Fig. 3A) were reduced immediately after the procedure (Fig. 3B). Five months after HIFU, the patient reported anal swelling and was diagnosed with a grade II hemorrhoid. Ten months after HIFU, the patient stopped breastfeeding and underwent aluminum potassium sulfate and tannic acid (ALTA) sclerotherapy [18]. Five years after the procedure, no prolapse was observed.

**Table 1:** Patient characteristics, treatment, and outcomes in high-intensity focused ultrasound rectoanal lifting for prolapsed hemorrhoids.

**Case**	**A (Case 1)**	**B (Case 2)**	**C (Case 3)**	**D (Case 4)**	**E (Case 5)**	**F (Case 6)**	**G (Case 7)**	**H (Case 8)**	**I (Case 9)**	**J (Case 10)**
**Patient characteristics**										
Sex/Age (years)/BMI (kg/m^2^)	Female/31/20	Female/31/22	Female/26/21	Female/33/19	Female/29/22	Female/54/18	Female/64/13	Female/60/25	Male/54/23	Male/55/24
Parity/Breastfeeding status	1/Exclusively	1/Exclusively	1/Exclusively	2/Mixed	1/Exclusively	0	2	0		
Period of disease	1 month	3 months	3 weeks	7 months	3 days	8 days	6 months	7 days	5 days	9 days
Surgical history (hemorrhoid)	Excision	ALTA			Thrombectomy					Excision
Goligher grade	**IV**	**IV**	**IV**	**IV**	**IV**	**IV**	**IV**	**IV**	**IV**	**IV**
Symptoms (excluding prolapse)	Anal pain	Anal pain	Anal pain	Anal pain	Anal pain	Anal pain	Anal pain	Anal pain	Anal pain	Anal pain
	Bleeding	Bleeding	Pruritus	Bleeding	Bleeding	Bleeding			Bleeding	
Comorbidities	Constipation	Constipation	Constipation	Constipation	Diarrhea	Constipation	Constipation	Diarrhea	Diarrhea	Hypertension
							Anorexia nervosa	Uterine cancer		
MRP/MSP (mmHg)		82/140	104/108	91/103	76/210	101/186	79/106	59/119	55/169	
**Treatment (HIFU-RAL)**										
Visit-to-treatment interval (days)	1	5	5	7	1	5	1	3	1	0
Anesthesia/LIS performed	None/None	Local/Yes	Local/Yes	Local/Yes	Local/Yes	Local/Yes	Local/Yes	None/None	Local/Yes	None/None
Cartridge type	Transvaginal	Transanal	Transanal	Transanal	Transanal	Transanal	Transanal	Transanal	Transanal	Transanal
Irradiation count M3.0/D4.5	23/23	0/90	0/90	90/90	90/90	45/90	90/90	45/90	90/90	45/90
Procedure duration (min)	12	11	9	15	16	11	15	12	20	14
**Outcomes**										
Prolapse recurrence/Time	Yes/5 months	None	None	None	None	Yes/21 days	None	None	None (2 months)	None (21 days)
Secondary treatment	ALTA					ALTA	ALTA		ALTA	Excision, ALTA
Treatment timing	10 months					21 days	3 months		3 months	21 days
Follow-up duration	5 years	9 days	19 days	2 months	2 months	1 year	4 months	3 months	9 months	10 months
Adverse events	None	None	None	None	None	None	None	None	None	None

Abbreviations: BMI, body mass index; ALTA, aluminum potassium sulfate and tannic acid; MRP, maximum resting pressure; MSP, maximum squeeze pressure; HIFU-RAL, high-intensity focused ultrasound rectoanal lifting; LIS, lateral internal sphincterotomy.

Case 2 involved a 31-year-old exclusively breastfeeding woman who had experienced a spontaneous delivery and had a history of hemorrhoid surgery (ALTA) 3 years ago (Table 1B). At 3 months postpartum, the patient presented with prolapsed hemorrhoids and difficulty in self-replacement. HIFU-RAL was performed 5 days later. Given the high MRP and anal stenosis, LIS and HIFU-RAL were performed. The prolapsed hemorrhoids (Fig. 3C) were reduced immediately after the procedure (Fig. 3D). On day 9, no hemorrhoidal prolapse was observed (Fig. 3E).

Case 3 involved a 26-year-old exclusively breastfeeding woman who had experienced a spontaneous delivery (Table 1C). The patient had developed a hemorrhoidal swelling 3 years ago, which had resolved spontaneously. She had developed painful prolapsed hemorrhoids immediately after delivery. These complications lasted for 3 weeks, and the patient presented with difficulty in self-replacement. As the patient declined medication, HIFU-RAL was performed 5 days later. Given the high MRP and anal stenosis, LIS and HIFU-RAL were performed. The prolapsed hemorrhoids (Fig. 3F) were reduced after the procedure (Fig. 3G). Nineteen days after the procedure, no prolapse was observed (Fig. 3H).

Case 4 involved a 33-year-old mixed breastfeeding woman who had experienced two spontaneous deliveries (Table 1D). The patient endured an anal prolapse persisting from the fifth month of her second pregnancy; however, she presented 1 month postpartum due to difficulty in self-replacement. HIFU-RAL was performed 7 days after obtaining consent from the obstetrician. Given the high MRP and anal stenosis, LIS and HIFU-RAL were performed. The circumferential prolapsed hemorrhoids (Fig. 3I) were reduced immediately after the procedure (Fig. 3J). Two months postoperatively, no prolapse was observed (Fig. 3K).

Case 5 involved a 29-year-old, exclusively breastfeeding woman who had experienced a spontaneous delivery (Table 1E). The patient improved with conservative hemorrhoid treatment 6 years before the current administration and underwent a thrombectomy 1 year before presentation. The patient presented with difficulty in self-replacement 3 days after experiencing prolapsed hemorrhoids. HIFU-RAL was performed on the following day. Given the high MRP, LIS, and HIFU-RAL were performed. The prolapsed hemorrhoids (Fig. 3L) were reduced immediately after the procedure (Fig. 3M). At 2 months postoperatively, prolapse reduction was maintained (Fig. 3N) with no recurrence.

**Figure 4:**
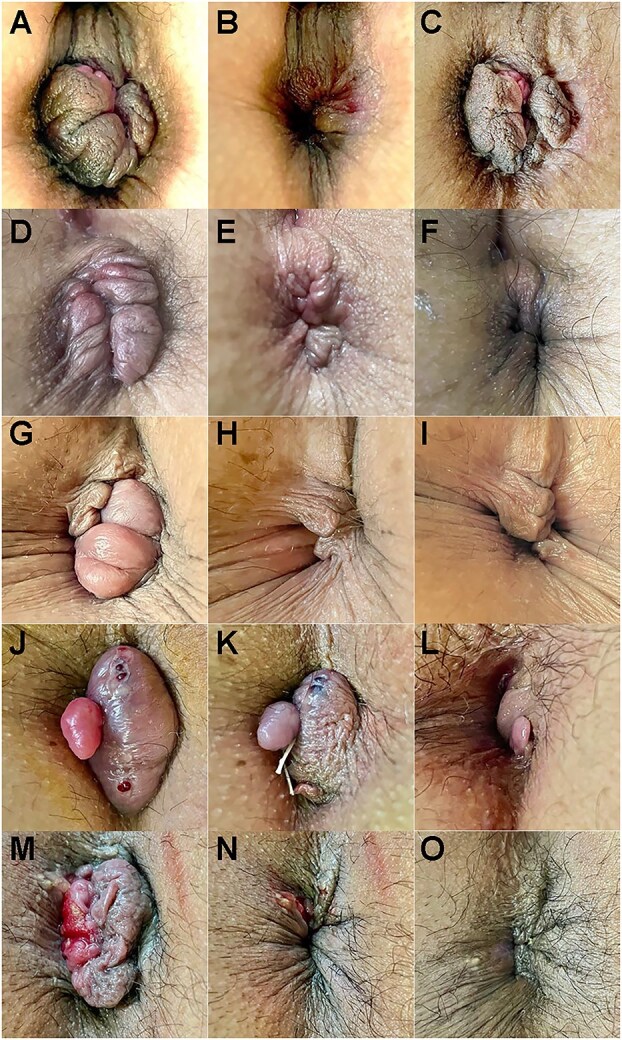
Longitudinal observation before and after HIFU rectoanal lifting (Cases 6–10). Top: Ventral. (A–C) Case 6 before and after HIFU (11 min) and 21 days later. (D–F) Case 7 before and after HIFU (15 min) and 3 months later. (G–I) Case 8 before and after HIFU (12 min) and 2 months later. (J–L) Case 9 before and after HIFU (20 min) and 2 months later. (M–O) Case 10 before and after HIFU (14 min) and 21 days later. The numbers in brackets indicate the time elapsed since the photograph was taken before HIFU.

Case 6 involved a 54-year-old woman with chronic constipation (Table 1F). The patient had left prolapsed hemorrhoids, which had been untreated for a year, and presented with an 8-day history of irreducible prolapsed hemorrhoids. After seeking minimally painful treatment, the patient underwent HIFU-RAL 5 days later. Given the high MRP, LIS and HIFU-RAL were performed. The prolapsed hemorrhoids (Fig. 4A) were reduced immediately after the procedure (Fig. 4B). However, on day 21, the prolapse recurred (Fig. 4C), requiring ALTA therapy. One year later, prolapsed hemorrhoids appeared on the right side, necessitating ligation excision.

Case 7 involved a 64-year-old woman who had experienced two spontaneous deliveries (Table 1G). The patient was diagnosed with anorexia nervosa and had a history of prolapsed hemorrhoids for 2 years, receiving conservative treatment intermittently. She experienced difficulty in self-replacement for 6 months, prompting presentation. Due to anorexia-induced significant body mass index (BMI) reduction, HIFU-RAL was performed the following day. Given the high MRP, LIS and HIFU-RAL were performed. The prolapsed hemorrhoids (Fig. 4D) were reduced immediately after the procedure (Fig. 4E). No prolapse was observed for 3 months (Fig. 4F); however, due to concerns regarding recurrence, ALTA was administered. No prolapse was observed for 1 month following ALTA.

Case 8 involved a 60-year-old woman who had undergone a total hysterectomy for uterine cancer 4 years ago and was undergoing chemotherapy (Table 1H). She had been diagnosed with prolapsed hemorrhoids 43 years ago, which had occasionally been managed with ointment. The patient presented with severe anal pain for 7 days. HIFU-RAL was chosen as a minimally invasive option and was performed 3 days later. The prolapsed hemorrhoids (Fig. 4G) were reduced immediately after the procedure (Fig. 4H). No re-prolapse was observed for 3 months (Fig. 4I), and chemotherapy was continued as planned.

Case 9 involved a 54-year-old delivery driver diagnosed with prolapsed hemorrhoids 3 years ago, which had improved with ointment treatment (Table 1I). Five days before the current admission, the patient developed prolapsed hemorrhoids, which protruded during defecation, making self-replacement difficult. To relieve pain quickly and resume work, the patient underwent HIFU-RAL the following day. The MRP was normal; however, rectal examination showed anal stenosis, requiring LIS. Prolapsed hemorrhoids and a large anal polyp were observed (Fig. 4J). The anal polyp was ligated, and HIFU-RAL immediately reduced the hemorrhoids (Fig. 4K). The patient resumed work the following day and experienced no prolapse for 2 months (Fig. 4L). ALTA was administered 3 months later for potential recurrence. No prolapse was observed 9 months post-HIFU-RAL.

Case 10 involved a 55-year-old fisherman who developed prolapsed hemorrhoids 3 months before presentation (Table 1J). Ventral prolapsed hemorrhoids were observed, and ligation excision was performed the following day. After returning to work, the patient developed left-sided prolapsed hemorrhoids on postoperative day 4, making self-replacement difficult. Symptoms persisted for 9 days, prompting a revisit. Due to work constraints, urgent excision was declined, and HIFU-RAL was performed on the same day. The prolapsed hemorrhoids (Fig. 4M) were reduced immediately after the procedure (Fig. 4N). The patient resumed work the following day. On postoperative day 21, the patient’s condition had improved from Goligher grade IV to III (Fig. 4O). On the same day, ligation excision and ALTA were performed. No recurrence was observed 10 months postoperatively.

## Discussion

To the best of my knowledge, this is the first case series on transanal HIFU for prolapsed hemorrhoid treatment. HIFU-RAL was performed in 10 patients, with a median time of 1.0 day (interquartile range (IQR): 1.0–4.5 days) from presentation, and a mean procedure duration of 13.5 ± 3.2 min. High adherence and safety were achieved without vital sign changes. Hemorrhoid reduction and early pain relief were observed immediately after treatment. HIFU-RAL was a viable option for patients avoiding excision due to surgical anxiety or work concerns (Cases 6, 9, and 10) and for those with high postoperative complication risks, where surgery was best avoided (Cases 7 and 8).

Macchi *et al*. reported that the thickness at the anal canal origin, where the rectal circular muscle (CM) transitions into the internal anal sphincter (IAS), was 2.7 ± 0.5 mm, while that of the longitudinal muscle (LM) measured 2.1 ± 0.3 mm [19]. Rociu *et al*. reported an IAS thickness of 2–3 mm and an LM thickness of approximately 2.5 mm [20]. Thus, in HIFU-RAL, the 3.0-mm cartridge acts on the CM transitioning into the IAS, while the 4.5-mm cartridge targets the LM. The irradiation area likely extends beyond the anal cushions to the rectal wall, given its 25 mm length and proximity to the dentate line. Following HIFU-RAL, hemorrhoids decreased immediately in our case studies, suggesting that thermal coagulation induces contraction of the CM, IAS, and LM, leading to upward displacement of the anal cushions and hemorrhoid shrinkage. LIS relieves pain after hemorrhoidectomy but may cause temporary FI [21]. LIS only enabled HIFU-RAL with a 23-mm cartridge in this study, with no pain or FI.

The incidence of hemorrhoids and anal fissures is high in late pregnancy and postpartum; with constipation, hemorrhoids, and anal fissures recognized as risk factors [22]. Cases 1–5 involved postpartum prolapsed hemorrhoids, which developed before or during pregnancy; four of which (80%) involved a history of constipation.

While pharmacological and surgical treatments are avoided during breastfeeding, persistent pain can negatively impact breast milk production and overall quality of life [23, 24]. HIFU-RAL effectively alleviates the anal pain associated with prolapsed hemorrhoids. During lactation, HIFU alone required no anesthesia, antibiotics, or analgesics, and no side effects were observed (Case 1). However, when combined with LIS, local anesthesia was required. In exclusively breastfeeding women, preprocedural measures, such as breast milk expression, are recommended for safety. Given the recurrence risk and limited long-term efficacy of HIFU-RAL, appropriate treatment should be considered after weaning.

HIFU-RAL was viable for patients avoiding excision due to surgical anxiety or work concerns (Cases 6, 9, and 10) and for those at high postoperative risk where surgery was best avoided (Cases 7 and 8). HIFU-RAL also prevented poor healing from anorexia-related BMI reduction (Case 7) and reduced the infection risk during chemotherapy, which was not interrupted (Case 8).

With the focus on sustainable development goals (SDGs) in healthcare, operating theaters, as the largest consumers of resources and CO₂ within hospitals, require improvements aligned with SDG 3.9 and SDG 12.5 [25]. HIFU-RAL supports these goals by enabling early hemorrhoid reduction, alleviating patient burden, enabling a swift return to work, and contributing to SDGs by reducing anesthesia, consumables, and medical waste. However, large-scale clinical trials and multicenter validations are essential to establish the effectiveness, safety, and long-term outcomes of HIFU-RAL.

In conclusion, HIFU-RAL is a minimally invasive treatment with shorter procedure times. In cases with insufficient initial response, additional treatment sessions can be administered. Moreover, combining HIFU-RAL with other minimally invasive therapies may enhance its effectiveness and broaden its applicability across a wider patient population.

## Data Availability

The data and materials that support the findings of this study are available from the corresponding author upon reasonable request.
